# No associations between type 1 diabetes and atopic dermatitis, allergic rhinitis, or asthma in childhood: a nationwide Danish case-cohort study

**DOI:** 10.1038/s41598-023-47292-5

**Published:** 2023-11-15

**Authors:** Anna Korsgaard Berg, Jannet Svensson, Jacob P. Thyssen, Bo Chawes, Claus Zachariae, Alexander Egeberg, Steffen Ullitz Thorsen

**Affiliations:** 1 Department of Pediatrics, Herlev and Gentofte Hospital, Herlev, Denmark; 2 Steno Diabetes Center Copenhagen, Copenhagen University Hospital, Borgmester Ib Juuls Vej 83, 2730 Herlev, Denmark; 3 Department of Clinical Medicine, Faculty of Health and Medical Sciences, University of Copenhagen, Copenhagen, Denmark; 4 Department of Dermatology and Venerology, Bispebjerg Hospital, København, Denmark; 5 Copenhagen Prospective Studies on Asthma in Childhood (COPSAC), Herlev and Gentofte Hospital, University of Copenhagen, Copenhagen, Denmark; 6 Department of Dermatology and Allergy, Herlev and Gentofte Hospital, Gentofte, Denmark; 7 Department of Clinical Immunology, Rigshospitalet, University of Copenhagen, Copenhagen, Denmark

**Keywords:** Epidemiology, Paediatric research

## Abstract

Studies examining the association between type 1 diabetes (T1D) and atopic diseases, i.e., atopic dermatitis, allergic rhinitis and asthma have yielded conflicting results due to different algorithms for classification, sample size issues and risk of referral bias of exposed cohorts with frequent contact to health care professionals. Using Danish national registries and well-established disease algorithms, we examined the bidirectional association between T1D and atopic diseases in childhood and adolescence using Cox Proportional Hazard regression compared to two different unexposed cohorts from a population of 1.5 million Danish children born from 1997 to 2018. We found no associations between T1D and atopic dermatitis, allergic rhinitis, or asthma (defined after age five). However, in multivariable analysis we found an increased risk of persistent wheezing (defined as asthma medication before age five) after T1D with an adjusted hazard ratio (aHR) of 1.70 [1.17–2.45]. We also identified an increased risk of developing T1D after persistent wheezing with aHR of 1.24 [1.13–1.36]. This study highlights similar risks of atopic diseases in children with T1D and of T1D in children with atopic disease after age of five years versus healthy controls. However, more research is needed to understand the possible early immunological effects of the link between persistent wheezing and T1D.

## Introduction

Type 1 diabetes (T1D) is characterized as an immune disease^1^ with increasing incidence worldwide^2^. T1D is often diagnosed in childhood and there are currently more than 1.5 million children and adolescents living with the condition^3^. Atopic diseases including atopic dermatitis, allergic rhinitis, and asthma are also characterized as immunologic-based diseases^4^ and are common in childhood.

A basic immunologic model involves differentiation of CD4 T-cells into two major subtypes; Th1 and Th2 cells which are determined based on their cytokine profile^5^. According to this model, T1D is considered mainly Th1-mediated whereas atopic diseases are predominately classified as Th2-mediated^6^. Based on this premise, an inverse association between the two disease groups would be expected due to opposing immunological phenotypes. On the other hand, T1D and atopic diseases have an overlap of genetic variation, especially in genes that are associated with pivotal immunological functions^7^. Observational studies of diverse quality due to study design limitations including small sample sizes, choice of control group, possibility of referral bias and differences in disease classification, have yielded heterogenic results with positive, inverse, and no associations^8–11^. Therefore, large, and well-designed complex studies are needed to clarify the bidirectional association between T1D, and the three atopic diseases. By linking the near complete and unique Danish registries containing diagnoses^12^ and prescriptions^13^, we had the opportunity to thoroughly examine the possible association between different algorithms for atopic diseases and T1D up to the age of 20 years in over 1.5 million Danish children born from 01–01–1997 to 31–12–2018.

Our primary aim was to investigate the association between T1D and the three atopic diseases: (i) atopic dermatitis, (ii) allergic rhinitis, and (iii) asthma using a bidirectional time-to-event analysis (also known as survival analysis).

## Methods

### Introduction to overall study design

In this prospective case-cohort study, we used register-data of both diagnoses (from admissions and out-patient clinics) and redeemed prescriptions of drugs to investigate the association of T1D and the three atopic diseases in both directions with time-to-event analysis. All our study cohorts were based on Danish citizens born from 01–01–1997 to 31–12–2018, excluding individuals with migration back and forth and children with the diagnosis of cystic fibrosis associated to a specific form of diabetes^14^. The period was chosen to ensure valid prescriptions for children (introduced in 1996) and end-of-follow-up was due to available register data. Data on medications includes only prescribed medication that was also redeemed^13^. Full reproducible algorithms for all disease criteria and further definitions can be found in Supplementary Methods.

### Disease algorithms

T1D was classified according to diagnosis code E10 and E14 shown to be highly valid in the pediatric age group^15^. Atopic dermatitis was defined by diagnosis code L20 proven to have high validity and thereby specificity^16^. Allergic rhinitis and asthma were (both as exposure and outcome) included in three different algorithms to ensure robustness of analysis and account for limitations of classification validity, but the most specific diagnosis-based algorithm was used in primary analysis for each disease group and the rest were used in the secondary analysis. For allergic rhinitis algorithm A was based on hospital diagnosis of rhinitis (J30, H101 and H104A) found with high specificity but low sensitivity by Leth-Møller et al*.*^17^, algorithm B was based on prescription data from the same author with higher sensitivity and lower specificity^17^ and algorithm C was based on a combination of diagnoses and prescription data using Henriksen et al.*’s* algorithm^18^ which was found to have much higher sensitivity but lower specificity^19^. Asthma was also defined by three different algorithms (A–C), although only suitable for children above 5 years^20^. Algorithm A was based on hospital diagnoses (J45, J450, J451, J458, J459, J469) which had been validated in children^21^ with very high specificity, algorithm B from Moth et al*.* was based on prescription data with high specificity^22^ and algorithm C was defined as inhaled corticosteroids and leukotriene antagonists as anti-asthmatic drugs, while children under 5 years of age fulfilling algorithm C were termed persistent wheezing. Clinical onset was defined as the first date of hospital contact or first date of redeemed prescription.

### Study cohorts

The exposed T1D-cohort consisted of all individuals in the study population diagnosed with T1D before the age of 20 years. The primary unexposed or control cohort consisted of ten random age- and sex-matched individuals without T1D for everyone in the exposed cohort. The secondary unexposed cohort consisted of all individuals in the study population with cerebral palsy (CP) to control for possible referral bias for T1D-cases which are frequently seen by health-care-professionals^23^. Individuals in both case and control cohorts with a diagnosis of any of the three atopic diseases (based on relevant diagnosis codes from algorithm A) prior to the start date of the follow-up period were excluded.

**Figure 1:**
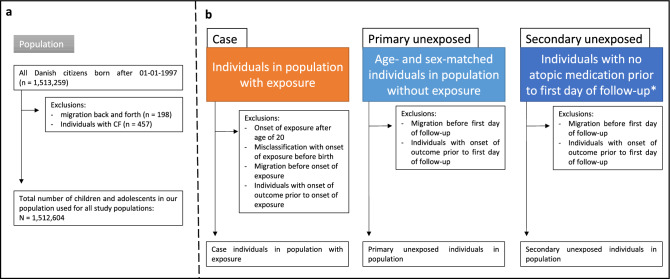
Flowchart for study cohorts. Panel a shows how the study population of 1,512,604 is selected and panel b shows the overall process of selecting the case-cohort, primary unexposed, and secondary unexposed cohort of each of the nine study cohorts. Specific numbers of individuals in each of the nine study cohorts are shown in respectively Table 1, Table 2, Supplementary Table S1 and Supplementary Table S2, and all flowcharts are shown in Supplementary Figure S2–S10. *Secondary unexposed cohort for the atopic-exposed cohort was taken from the primary unexposed cohort to ensure correct study start and for type 1 diabetes-exposed cohort consisted of individuals with cerebral palsy excluding individuals with both type 1 diabetes and cerebral palsy as well.

The three primary exposed atopic cohorts consisted of individuals diagnosed with atopic dermatitis, allergic rhinitis (in algorithm A) or asthma (in algorithm A) before the age of 20. The five secondary exposed atopic cohorts consisted of individuals diagnosed with allergic rhinitis or asthma in the two less specific algorithms (B and C) before the age of 20 or persistent wheezing defined by algorithm C up to the age of 5. The primary unexposed cohorts consisted of up to ten random age- and sex-matched individuals without the specific atopic disease with same algorithm as the exposed cohort. The secondary unexposed cohort consisted of all matched individuals with no atopic medication prior to the start date of the follow-up period (see Fig. 1). Individuals with a diagnosis of T1D prior to the start date of the follow-up were excluded from both case and control cohorts.

### Statistical analyses

Cox proportional hazard regression models, hereinafter referred to as ”Cox regression”, were used for our main analyses to model the relationship between the survival time until outcome (time-to-event data) and one or more predictor or exposure variables with corresponding Kaplan–Meier plots. Results are presented with both incidence rates (IR) for each cohort and hazard ratio’s (HRs) with 95% confidence intervals (95% CI). Model control was conducted to examine the Cox regression model assumptions of proportional hazards and linearity. If the latter was not fulfilled, we converted the continuous variable into a categorical variable. P-values from primary and secondary analyses were corrected for multiple testing using the Benjamini–Hochberg method for each disease-specific question (family-wise correction). However, raw p-values are also presented. In addition, no power calculation was performed because both exposure and outcome variables already existed in the registry, and we therefore refer to the presented 95% confidence intervals (CI). Moreover, we expected enough power for our a priori defined primary and secondary research questions^24^. For the exposed T1D-cohort, three outcomes were analyzed: (i) time to onset of atopic dermatitis or censoring; (ii) time to allergic rhinitis or censoring and (iii) time to onset of asthma or censoring. For all exposed atopic cohorts, that is atopic dermatitis, allergic rhinitis, or asthma, the analyzed outcome was time to onset of T1D or censoring. Censoring was defined as death, migration, or end of follow-up. End of follow-up was defined as age of 20 years or end of registry 31–12–2018. A gap of at least three months between clinical onset of exposure and outcome was defined to reduce referral bias and outcome events after exposure prior to that gap were ignored. The primary analyses included only the diagnosis-based criteria for atopic diseases (algorithm A for both asthma and rhinitis), the most specific, although less sensitive, algorithms for both exposure and outcomes classification. The secondary analyses included the other algorithms for rhinitis, asthma, and persistent wheezing with more cases (and therefore power) but less strict algorithms with greater risk of misclassification.

The following covariates were chosen a priori based on the literature and directed acyclic graphs (DAGs): sex, age, child delivered by caesarian section, prematurity, season of birth and family history of the outcome disease. Both results from unadjusted and adjusted regression models are reported. All definitions are found in Supplementary Methods and the two DAGs are found in Supplementary Figure S1.

Several sensitivity- and post-hoc analyses including food allergy based on diagnosis code and severe food allergy with prescription on adrenaline were conducted to increase the robustness of conclusions which are further described in Supplementary Methods.

SAS statistical software version 9.4 was used for all data management steps, and all analyses were performed using the statistical software package R, version 4.0.3 (the R Foundation for Statistical Computing, Vienna, Austria).

### Study approvals

All study data was based on available registries from Statistics Denmark. The study was approved by the Danish Data Protection Agency and subsequently registered at the Capital Region’s Inventory (*Videnscenter for Dataanmeldelser* with the number VD-2018–286). The study was performed in agreement with the Declaration of Helsinki^25^ and according to Danish legislation (Danish Law: *Lov om videnskabsetisk behandling af sundhedsvidenskabelige forskningsprojekter*, § 14, stk. 2) no further consent or ethical approval was required for registry-based studies as this study with no use of biological material and no direct contact to participants.

## Results

### Baseline characteristics of cohorts

Figure 1 shows the flowchart for the different study cohorts as described in the method section. The full study population included all Danish citizens born after 01–01–1997, totaling 1,513,259 individuals from which the nine different study cohorts are extracted with exposures of T1D, atopic dermatitis, three algorithms of allergic rhinitis, three algorithms of asthma and one of persistent wheezing as shown in Supplementary Figure S2–S10.

Table 1 shows basic demographics of the T1D-exposed cohort (n = 4111) and its unexposed cohorts of random age- and sex-matched individuals (n = 41,110) and individuals with cerebral palsy (n = 4546), respectively. Different distributions are evident for the T1D-exposed and cerebral palsy cohorts regarding preterm birth, born by caesarian section, and at least one parent with T1D. Mean age for T1D onset was 8.9 years. Table 2 shows basic demographics of the three primary atopic-exposed cohorts (based on the specific A-algorithms) including 22,612 with atopic dermatitis (mean age at inclusion 3.8 years), 18,516 with allergic rhinitis (mean age 6.4 years) and 36,214 with asthma (mean age 9.3 years).

**Table 1:** Basic demographics of T1D-exposed cohort and unexposed cohorts.

	T1D-case (N = 4111)	Primary unexposed* (N = 41,110)	Secondary unexposed† (N = 4546)
Sex (Female)	2000 (48.7%)	20,000 (48.7%)	1924 (42.3%)
Age at inclusion in study (Mean (SD)) in years	8.86 (4.60)	8.86 (4.60)	6.51 (4.77)
Preterm Birth	122 (3.0%)	911 (2.2%)	898 (19.8%)
Born by C-section	812 (19.8%)	6931 (16.9%)	1603 (35.3%)
Season of birth			
Autumn	1000 (24.3%)	10,000 (24.3%)	1141 (25.1%)
Spring	1108 (27.0%)	11,075 (26.9%)	1142 (25.1%)
Summer	1067 (26.0%)	10,670 (26.0%)	1193 (26.2%)
Winter	936 (22.8%)	9365 (22.8%)	1070 (23.5%)
Siblings with atopic dermatitis	62 (1.5%)	580 (1.4%)	67 (1.5%)
Siblings with rhinitis	50 (1.2%)	578 (1.4%)	48 (1.1%)
Siblings with asthma	123 (3.0%)	1159 (2.8%)	136 (3.0%)
At least one parent with T1D	423 (10.3%)	711 (1.7%)	104 (2.3%)

*Abbreviations* SD, standard deviation, C-section, caesarian section, T1D, Type 1 Diabetes.

*Ten random age- and sex-matched individuals per case.

^†^The secondary unexposed cohort of individuals with cerebral palsy was compared to the case-cohort of 4084 since individuals with both cerebral palsy and T1D were subsequently excluded from both cohorts.

**Table 2:** Basic demographics of primary atopic-exposed cohort and unexposed cohorts.

	Atopic dermatitis case (N = 22,612)	Primary unexposed* (N = 226,120)	Secondary unexposed (N = 79,615)
Sex (Female)	9977 (44.1%)	99,770 (44.1%)	32,780 (41.2%)
Age at inclusion in study (Mean (SD))	3.84 (4.20)	3.84 (4.20)	2.18 (2.59)
Born by C-section	4565 (20.2%)	40,170 (17.8%)	14,212 (17.9%)
Preterm birth	769 (3.4%)	7125 (3.2%)	2219 (2.8%)
Season of birth			
Autumn	5987 (26.5%)	59,870 (26.5%)	21,022 (26.4%)
Spring	5500 (24.3%)	55,000 (24.3%)	19,552 (24.6%)
Summer	5593 (24.7%)	55,930 (24.7%)	19,360 (24.3%)
Winter	5532 (24.5%)	55,320 (24.5%)	19,681 (24.7%)
At least one parent with T1D	405 (1.8%)	3598 (1.6%)	1353 (1.7%)

	Allergic rhinitis† case (N = 18,516)	Primary unexposed* (N = 185,160)	Secondary unexposed (N = 43,017)
Sex (Female)	6997 (37.8%)	69,970 (37.8%)	16,247 (37.8%)
Age at inclusion in study (Mean (SD))	6.42 (4.19)	6.42 (4.19)	4.37 (3.32)
Born by C-section	3643 (19.7%)	31,934 (17.2%)	7462 (17.3%)
Preterm Birth	518 (2.8%)	4547 (2.5%)	937 (2.2%)
Season of birth			
Autumn	4334 (23.4%)	43,340 (23.4%)	9881 (23.0%)
Spring	4945 (26.7%)	49,450 (26.7%)	11,792 (27.4%)
Summer	4619 (24.9%)	46,190 (24.9%)	10,451 (24.3%)
Winter	4618 (24.9%)	46,180 (24.9%)	10,893 (25.3%)
At least one parent with T1D	369 (2.0%)	3162 (1.7%)	798 (1.9%)

	Asthma§ case (N = 36,214)	Primary unexposed* (N = 362,140)	Secondary unexposed (N = 55,296)
Sex (Female)	14,761 (40.8%)	147,610 (40.8%)	22,537 (40.8%)
Age at inclusion in study (Mean (SD))	9.32 (3.60)	9.32 (3.60)	8.04 (2.86)
Born by C-section	7277 (20.1%)	60,376 (16.7%)	8627 (15.6%)
Preterm Birth	1156 (3.2%)	7385 (2.0%)	781 (1.4%)
Season of birth			
Autumn	8799 (24.3%)	87,985 (24.3%)	13,083 (23.7%)
Spring	9305 (25.7%)	93,032 (25.7%)	14,443 (26.1%)
Summer	9382 (25.9%)	93,825 (25.9%)	14,522 (26.3%)
Winter	8728 (24.1%)	87,298 (24.1%)	13,248 (24.0%)
At least one parent with T1D	759 (2.1%)	6379 (1.8%)	1048 (1.9%)

Abbreviations: SD, standard deviation, C-section, caesarian section, T1D, Type 1 Diabetes.

*Ten random age- and sex-matched individuals per case.

^†^Primary allergic rhinitis cases were based on model A (diagnosis code).

^§^Primary asthma cases were based on model A (diagnosis code).

Supplementary Table S1 and Supplementary Table S2 include baseline characteristics for study cohorts based on the other algorithms (B and C) for asthma, persistent wheezing and allergic rhinitis, and their unexposed cohorts.

### T1D as exposure for atopic disease

**Figure 2:**
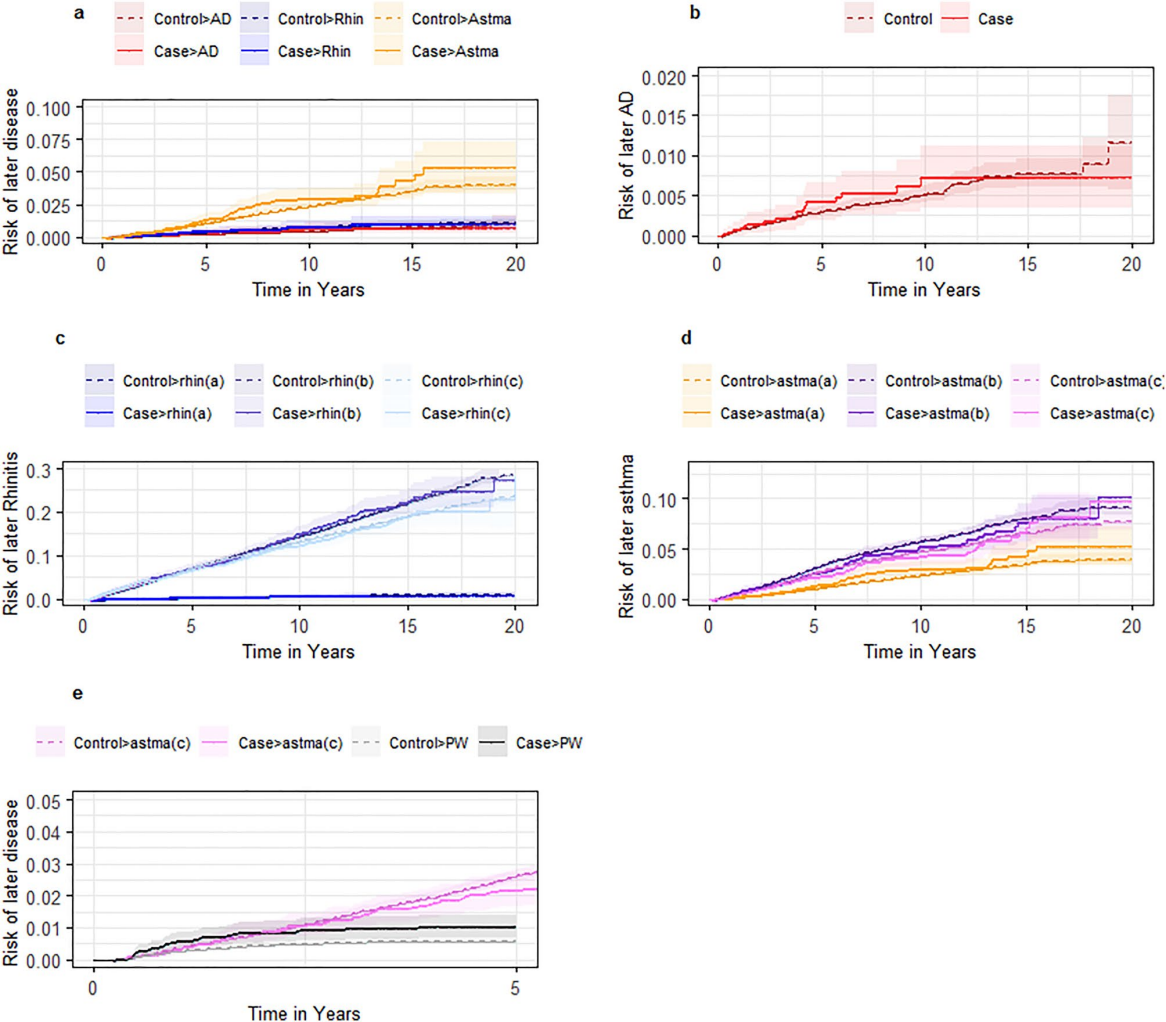
Risk of atopic disease after onset of type 1 diabetes. Abbreviations: AD: Atopic dermatitis. PW: Persistent wheezing. Rhin: allergic rhinitis. All panels in Fig. 2 show Kaplan–Meier curves for the risk of later atopic disease after onset of Type 1 Diabetes compared to the primary unexposed cohort of healthy controls. Panel a shows the risk of later atopic dermatitis, allergic rhinitis, or asthma, all defined by the diagnoses criteria (algorithm A). Panel b shows the risk of later atopic dermatitis. Panel c shows the risk of later allergic rhinitis defined by the three algorithms for allergic rhinitis (A, B and C). Panel d shows the risk of later asthma defined by the three algorithms for asthma (A, B and C) after age of five. Panel e show the risk of later asthma defined the algorithm C for children up to age five and the risk of later persistent wheezing by same algorithm C though only up to age five years and limiting the follow-up to the first five years after onset of type 1 diabetes.

We found no associations between T1D-exposed individuals and risk of atopic dermatitis, rhinitis, or asthma (after age of 5 years) in our adjusted Cox regressions irrespective of algorithms for the atopic diseases or choice of control cohort (Fig. 2, Table 3, and Supplementary Table S3). However, we found an increased risk of persistent wheezing in the T1D-exposed cohort: adjusted HRs (aHRs) of 1.70 [95% CI 1.17–2.45], p_corrected_ = 0.041 compared to the primary unexposed cohort, but no difference was found in the adjusted analyses when compared to individuals with cerebral palsy (Supplementary Table S3). Incidence rates of the different atopic diseases in different algorithms varied from 0.62–41.68 per 1000 person-years (Table 3 and Supplementary Table S3).

**Table 3:** Effects of Type 1 Diabetes on later atopic disease.

	IR_A_ [95%CI]	IR_B_ [95%CI]	HR [95% CI]	P_raw_	P_corr_	N	Events
Risk of atopic dermatitis							
Unadjusted model	0.74 [0.47;1.17]	0.62 [0.53;0.73]	1.18 [0.71;.95]	0.520	1.000	44,000	160
Adjusted model§			1.08 [0.64;1.80]	0.784	1.000	42,102	159
Risk of allergic rhinitis							
Unadjusted model A	0.94 [0.63;1.40]	0.86 [0.75;0.98]	1.01 [0.64;1.58]	0.978	1.000	44,004	228
Adjusted model§ A			1.00 [0.64;1.56]	0.988	1.000	42,106	222
Unadjusted model B	30.32 [28.13;32.68]	29.74 [29.03;30.46]	1.02 [0.91;1.14]	0.750	1.000	40,611	3801
Adjusted model§ B			0.97 [0.87;1.08]	0.552	1.000	38,744	3736
Unadjusted model C	38.60 [36.04;41.34]	37.56 [36.74;38.39]	0.96 [0.85;1.08]	0.525	1.000	38,747	3342
Adjusted model§ C			0.92 [0.82;1.04]	0.180	1.000	36,904	3263
Risk of asthma							
Unadjusted model A	3.25 [2.62;4.04]	2.43 [2.24;2.63]	1.23 [0.96;1.57]	0.097	0.398	43,981	654
Adjusted model§ A			1.17 [0.92;1.50]	0.208	0.555	42,083	641
Unadjusted model B	13.17 [11.80;14.71]	11.81 [11.39;12.26]	0.88 [0.74;1.06]	0.184	0.555	42,463	1538
Adjusted model§ B			0.86 [0.71;1.03]	0.099	0.398	40,583	1499
Unadjusted model C	11.11 [9.86;12.52]	9.89 [9.51;10.30]	0.94 [0.77;1.14]	0.512	1.000	42,682	1281
Adjusted model§ C			0.91 [0.75;1.10]	0.336	0.768	40,799	1259
Unadjusted Persistent wheezing	2.52 [1.80;3.53]	1.43 [1.25;1.64]	1.71 [1.19;2.48]	0.004	0.041	39,791	232
Adjusted§ Persistent wheezing			1.70 [1.17;2.45]	0.005	0.041	37,933	230

Raw incidence rates (IR) shown per 1000 person-year for respectively T1D-exposed cohort (A) and the primary unexposed cohort as control cohort (B), hazard ratio (HR) from Cox regressions is based on exposed case cohort compared to the primary unexposed cohort (age- and sex matched individuals). The raw p-values are shown as P_raw_, where P_corr_ are familywise corrected by Benjamini-Hochberg.

^§^Adjusted models are adjusted for age group, sex, born by caesarian section, prematurity, season of birth and relevant family history (sibling with asthma for models of risk of asthma etcetera).

### Atopic disease as exposure for T1D

**Figure 3:**
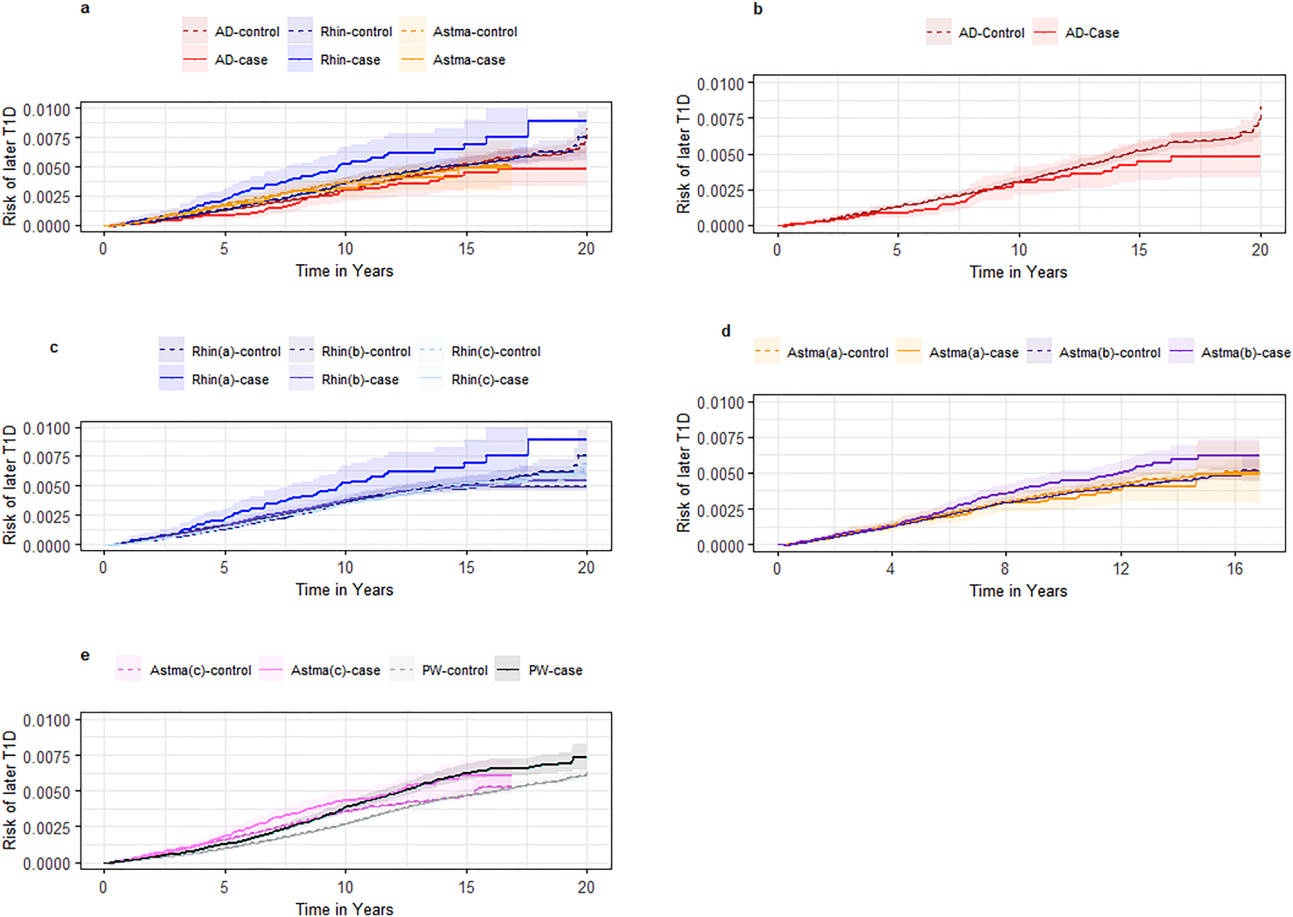
Risk of type 1 diabetes after onset of atopic disease. Abbreviations: AD: atopic dermatitis, PW: Persistent wheezing. Rhin: allergic rhinitis. T1D: Type 1 Diabetes. All panels in Fig. 3 show the Kaplan–Meier curves for the risk of later Type 1 Diabetes (T1D) after onset of atopic disease compared to the primary unexposed cohort of healthy controls. Panel a shows the exposed cohorts of individuals with atopic dermatitis, allergic rhinitis, or asthma, where all exposures were based on diagnoses (algorithm A). Panel b shows the exposed cohorts of individuals with atopic dermatitis. Panel c shows the exposed cohorts of individuals with allergic rhinitis defined by the three algorithms for rhinitis (A, B and C). Panel d shows the exposed cohorts of individuals with asthma in algorithm A and B after age of five years. Panel e show the exposed cohort of individuals with asthma in algorithm C respectively after age of five years (termed Astma(c)) and up to age of five (termed persistent wheezing).

Similarly, our adjusted Cox regressions revealed no significant associations between the atopic dermatitis-, allergic rhinitis-, or asthma- (after age of five years) exposed cohorts and the risk of developing T1D, irrespective of the algorithms used to define atopic diseases or choice of control cohort (Fig. 3**, **Table 4, and Supplementary Table S4). Positive associations were found between an increased risk of developing T1D after having allergic rhinitis in algorithm A and asthma in models B and C before correction for multiple comparisons (Table 4 and Supplementary Table S4). There was an increased risk of developing T1D in the persistent wheezing-exposed cohort with aHR [95%CI] of 1.24 [1.13–1.36], p_corrected_ < 0.001 compared to random age- and sex-matched controls. A similar trend was observed in comparison to the secondary control cohort aHR [95% CI] of 1.15 [1.03–1.28], p_corrected_ = 0.074. Incidence rates of later T1D varied from 0.27–0.71 per 1000 person-years (Table 4 and Supplementary Table S4).

**Table 4:** Effects of atopic diseases on later Type 1 Diabetes.

	IR_A_ [95%CI]	IR_B_ [95%CI]	HR [95% CI]	P_raw_	P_corr_	N	Events
Atopic dermatitis and risk of type 1 diabetes							
Unadjusted model	0.27 [0.20;0.35]	0.40 [0.37;0.43]	0.82 [0.61;1.10]	0.183	0.366	244,125	634
Adjusted model§			0.79 [0.59;1.06]	0.122	0.366	240,302	631
Allergic rhinitis and risk of type 1 diabetes							
Unadjusted model A	0.48 [0.38;0.60]	0.51 [0.47;0.54]	1.41 [1.10:1.80]	0.007	0.083	201,116	568
Adjusted model§ A			1.35 [1.05;1.72]	0.019	0.113	197,190	566
Unadjusted model B	0.36 [0.33;0.40]	0.70 [0.67;0.73]	1.01 [0.90;1.13]	0.849	1.000	720,143	1523
Adjusted model§ B			0.96 [0.86;1.08]	0.512	1.000	702,556	1512
Unadjusted model C	0.34 [0.32;0.37]	0.53 [0.51;0.56]	1.01 [0.92;1.12]	0.804	1.000	670,226	1725
Adjusted model§ C			0.96 [0.87;1.06]	0.443	1.000	655,475	1717
Asthma and risk of type 1 diabetes							
Unadjusted model A	0.35 [0.28;0.44]	0.71 [0.68;0.75]	0.92 [0.73;1.17]	0.497	0.994	387,989	873
Adjusted model§ A			0.89 [0.70;1.12]	0.319	0.730	378,564	866
Unadjusted model B	0.43 [0.38;0.49]	0.62 [0.60;0.65]	1.23 [1.07;1.40]	0.003	0.014	613,722	1503
Adjusted model§ B			1.17 [1.03;1.34]	0.020	0.065	598,904	1493
Unadjusted model C	0.42 [0.37;0.48]	0.61 [0.58;0.64]	1.21 [1.05;1.40]	0.010	0.039	524,786	1307
Adjusted model§ C			1.16 [1.00;1.34]	0.048	0.128	511,838	1297
Unadjusted model Persistent wheezing	0.38 [0.35;0.41]	0.31 [0.30;0.33]	1.30 [1.18;1.43]	< 0.001	< 0.001	855,780	2625
Adjusted§ Persistent wheezing			1.24 [1.13;1.36]	< 0.001	< 0.001	844,423	2618

Raw incidence rates (IR) shown per 1000 person-year for respectively the exposed cohort (A) and the primary unexposed cohort as control cohort (B), hazard ratio (HR) from Cox regressions is based on exposed case cohort compared to the primary unexposed cohort (age- and sex matched individuals). The raw p-values are shown as P_raw_, where P_corr_ are familywise corrected by Benjamini-Hochberg.

^§^Adjusted models are adjusted for age group, sex, born by caesarian section, prematurity, season of birth and relevant family history (parents with type 1 diabetes).

### Sensitivity analyses

Results from sensitivity post-hoc analyses are shown in Supplementary Table S5. (i) Too few cases with both T1D and atopic dermatitis were available to meaningfully examine the association with later risk of rhinitis and asthma. (ii) Neither mild atopic dermatitis or severe atopic dermatitis influenced the risk of developing T1D. (iii) Similarly, no differences were found in the risk of developing T1D depending on whether diagnosis of atopic dermatitis was made by a dermatologist or not. (iv) Atopic dermatitis, defined as one prescription of steroid lotion, increased the risk of developing T1D with aHR [95%CI] of 1.17 [1.09–1.26], p < 0.001, although Cox regression model assumptions were not fulfilled from T1D to atopic dermatitis with this definition of atopic dermatitis, the log-rank-test and Kaplan–Meier plots revealed a significant positive association between T1D and subsequent atopic dermatitis. Atopic dermatitis, defined by the algorithm by Henriksen *et al*^18^, showed a positive association between T1D and atopic dermatitis with aHR [95%CI] of 1.22 [1.05–1.40], *p* = 0.008, but not vice versa i.e., atopic dermatitis to T1D. (v) Neither food allergy or severe food allergy as exposures or outcomes were associated to T1D. (vi) The stratification of the three asthma-cohorts in T2-high or T2-low phenotypes did not result in any significant associations with the development of T1D.

## Discussion

In this large, population-based, prospective, case-cohort study, we found no clear associations between T1D and the three atopic diseases: atopic dermatitis, allergic rhinitis, or asthma after taking different algorithms of atopic diseases into account. However, we found an increased risk of persistent wheezing in those with early-onset T1D compared to a healthy control cohort. Furthermore, we detected a significant positive association in risk of developing T1D after persistent wheezing.

The increased risk of persistent wheezing after T1D diagnosis may be caused by an increased risk of upper airway infection caused by a pronounced inflammatory response and/or compromised immune system after developing early-onset T1D. Our criteria for persistent wheezing was based on drug use, and is therefore in agreement with the finding of more frequent use of asthma medication the first year after T1D diagnosis^26^, where levels of inflammation are higher as long as beta cells still exist^27^. Another explanation could be the referral bias of individuals with T1D having more frequent contact with health-care-professionals which was supported by the finding of similar prescriptions of asthma medications compared to individuals with cerebral palsy who were frequently followed-up. However, shared risk factors for cerebral palsy and persistent wheezing such as prematurity and C-section may also explain the lack of association when cerebral palsy individuals were used as control cohort, despite having adjusted for it in our multivariable analysis. No significant associations were found for the other asthma-algorithms after T1D, indicating that increased use of asthma medication accounts only in the first 5 years of life and do not increase the risk of asthma-diagnosis. Use of asthma medication in the first 5 years of life increased the risk of T1D throughout childhood, which was also associated with a greater tendency in the use of the same asthma medication after the age of 5 years, albeit not statistically significant after correction for multiple testing. An explanation for the association could be the increased susceptibility towards systemic metabolic adverse effects seen with corticosteroid inhalations in early life^28^, acting either as a trigger for islet-specific autoimmunity^1^, influencing insulin resistance or lower clearance of viral infections associated with T1D^29^. Similarly, the sensitivity analyses revealed an increased risk of developing T1D after the use of one prescription of steroid lotion, which may also induce adverse metabolic effects^30^. Another possibility is that the phenotype of persistent wheezing has an alternative inflammatory profile than that seen with classical asthma, a notion supported by the finding that those who persistently wheezed have low levels of eosinophile inflammation^31^. According to that framework, it could be queried as to whether persistent wheezing is at all characterized by Th2 inflammation. However, unadjusted incidence rates were quite comparable, thereby limiting the clinical relevance.

Different atopic disease algorithms prevent fair comparability between studies^8,33^. This is particularly evident in studies where diseases are classified as outcomes and exposures where a high validity of included cases is found to be more important than including many cases^24^. A meta-analysis of heterogenic cross-sectional studies on T1D and all three atopic diseases found no differences in the risk of atopic dermatitis or rhinitis with T1D but an inverse risk of asthma in T1D. These findings were, however, only apparent in studies defined in meta-analysis of adequate comparable design, highlighting the influence of design on conclusions^8^. Indeed, two recent reviews found an increased risk of T1D after asthma with HR of ~ 1.3 but without an association between T1D and the later development of asthma^11,34^.

The association between asthma and T1D has been studied in several other Scandinavian registry-based studies. A Swedish study found no association between T1D and asthma but, similar to our findings, noted a small increased risk of T1D after asthma with aHR of 1.17 [1.07–1.28]^35^. Both a Finnish study^9^ and a newly published Danish study^36^ found clear inverse associations between T1D and asthma with HRs of 0.70 [0.59–0.84] and 0.52 [0.31–0.68], respectively. Both studies also observed an increased risk of developing T1D after asthma^9,36^, with similar effect sizes to ours, albeit we used correction for multiple comparisons. Liljendahl et al*.* used the same registry database as us, but their definitions of asthma, age-groups, and control cohort differed considerably from ours, providing likely explanation for some of the differences in our conclusions^36^. Geographically, differences may exist. Indeed, a Chinese study found a HR of 1.34 [1.11–1.62] between T1D and the risk of developing asthma^37^ which contrasts both our and other Scandinavian studies^9,36^. Similarly, in a Taiwanese study, researchers noted an increased risk of atopic dermatitis after T1D diagnosis with aHR [95% CI] of 1.76 [1.29–2.39]^10^ which despite similar definitions, is in clear conflict to our results.

However, null findings may also be a consequence of mechanisms of both positive and inverse associations e.g., the net-sum of genetic, immunological, and environmental factors, in accordance to Rothman’s sufficient cause model^38^. A possible explanation for the presence of an inverse association is the type of immune inflammation being categorized as either type 1 (for T1D) or type 2 (for atopic diseases)^5^ when persistent wheezing (especially on viral cause) could be considered as type 1 rather than type 2. Some of the positive associations could be explained by shared genetic and environmental factors^35,39^. Furthermore, gut microbiota is considered an important component of proper immune system development^40^. Though the Th1/Th2 pattern is easy to understand, it is too simplistic since many new Th cell subsets have been discovered. Indeed, Th17 and regulatory T-cell subsets have been identified as important mediators of both atopic and autoimmune diseases^41^, leading to potentially many phenotypes of the same disease. Recently, it has been put forth that T1D includes different subtypes, depending on both clinical onset, different autoantibodies, and clinical phenotypes^29^. Similarly, asthma is thought to consist of both allergic and non-allergic asthma. In this theory, non-allergic asthma is considered to include autoimmune properties^42^, and differences in classical asthma and persistent wheezing exist. In our study, we were unable to distinguish these unclearly defined subtypes of diseases, rather, our sensitivity analyses tried to overcome difference in the severity and validity of the atopic part. To that end, we noted no differences among T2-high or T2-low asthma, comorbidities with food allergies or mild and severe atopic dermatitis. The classical “atopic march” of first atopic dermatitis, followed by asthma and then rhinitis is also a simplification, being challenged by machine-learning approaches of population-based-cohorts showing only occurrence in 3–7% of the population^43^.

Some of the strengths of this study are the large sample size compared to many other observational studies and the inclusion of a national population of children and adolescents with up to over two decades of follow-up time. Another strength is that we investigated not only one of the atopic diseases compared to T1D, but three of them in both directions with different algorithms for definitions. However, a limitation is the validity of our registry, since whilst the Danish registries are highly valid^44,45^, they only reflect the validity of the data included within. For diagnosis, only hospital contact can be included, which is not a problem with T1D which is only followed in specialized out-patient clinics at hospitals but might be for atopic diseases for which mild cases are followed by general practitioners. Therefore, some misclassification is expected for mild allergic cases when only diagnoses are included. The algorithms that also includes information from prescription registers solve this problem at the cost of specificity. Hence, individuals with a hospital code of atopic disease would be assumed as having a more severe disease or comorbidity. The latter could be a potential referral bias in our study, resulting in more atopic diagnoses or prescriptions in individuals with T1D compared to healthy individuals. This was the rationale behind the secondary unexposed cohort consisting of cerebral palsy, which also modified the conclusion. The drug data contains all drugs that have been prescribed and collected from all pharmacies in Denmark. Thus, validity is only limited when using drug data as proxy for disease when drugs are unspecific for that disease like medications for atopic dermatitis. However, drug data was not available before 1997, which limited our follow-up time. Another limitation was the natural age-related time-course of the diseases, which therefore limited the ability to find associations (type 2 error) since for instance atopic dermatitis typically have early onset. Further, we predominately excluded the mildest forms of atopic disease since antihistamines and topical hydrocortisone can be purchased over the counter and therefore are not captured in the registries. Lastly, our comprehensive study approach and our largely uniform results is much needed in a field of inconsistent results across studies. We decided in this study to balance the risk of type 1 and type 2 errors by using Benjamini–Hochberg corrections for multiple comparisons and interpreted results in that light^46^.

Taken together, no strong associations were found between atopic diseases and T1D, which is also reflected in the comparable incidence rates even for the strongest association between persistent wheezing after T1D in our results. In clinical practice, we must be aware that young individuals before the age of 5 with T1D use more asthma medication than healthy controls, but not more than individuals with cerebral palsy, when adjusting for potential confounders. More investigations are needed to understand whether the inflammatory response is truly increased or whether it is simply a product of patients being more prone to receiving prescriptions, here clinical studies of patients are needed. Similarly, studies investigating similarities between the underlying inflammation of persistent wheezing seen in preschool children andT1D or if susceptibility to viral infections is more important, are warranted. Moreover, potential adverse metabolic systemic effects of inhaled corticosteroids in preschool children may contribute to T1D risk, although the effect size seems limited. This also emphasizes that both shared and distinct features for atopic and autoimmune diseases, or at least T1D, exist.

In conclusion, this nationwide, bidirectional, registry-based study shows that there was no obvious association between the three atopic diseases and T1D, but that T1D was associated bidirectional with early childhood persistent wheezing.

### Supplementary Information

Supplementary Information.

## Data Availability

The data set for this study was made available from Statistics Denmark through a certain license and access, which make it impossible for the author group to make the data available for third parties. The data are though available upon request to Statistics Denmark if permission can be obtained.

## Abbreviations

aHR: Adjusted hazard ratio
ICD-10: International Classification of Diseases and Related Health Problems version 10
T1D: Type 1 Diabetes
