# Supplementary material for: No associations between type 1 diabetes and atopic dermatitis, allergic rhinitis, or asthma in childhood: a nationwide Danish case-cohort study
Source: Sci Rep. 2023 Nov 15;13:19933. doi: 10.1038/s41598-023-47292-5 (PMC10652009; doi:10.1038/s41598-023-47292-5)
Supplement: Supplementary file 1 — Supplementary Information. [file 41598_2023_47292_MOESM1_ESM.pdf]

Supplementary Material

Supplementary methods

Disease algorithms

T1D

T1D was defined by using the Danish National Patient Registry and the ICD-10 code: DE10, all other DE10x and DE14 which had been validated in a pediatric age group using the Danish Registry of Childhood and Adolescent Diabetes[15]. Clinical onset of T1D was defined as the first hospital contact with the diagnosis-code. For T1D in parents only DE10, and all other DE10x was used.

Atopic dermatitis

Atopic dermatitis was defined by using the Danish National Patient Register and the ICD-10 code: DL20. The validity of the atopic dermatitis-diagnosis in Danish registries have been proven high[16] and most medication is not disease-specific explaining why algorithms with prescriptions are not included to get a more sensitive algorithm. Clinical onset or progression of atopic dermatitis was defined as the first hospital contact with the diagnosis-code.

Allergic rhinitis

Allergic rhinitis is defined based on three different algorithms:

- A: based on a hospital diagnosis of allergic rhinitis (shown to yield high specificity (1.00) although low sensitivity (0.01) in adults[17]) based on ICD-10-codes J30, H101 and H104A.
- B: based on prescription data (showing specificity on 0.94 and sensitivity on 0.21 in adults[17]:
    - o At least two prescriptions of oral/nasal/ophthalmic antihistamine or intranasal corticosteroids with the drug codes: S01GA51, S01GX02, S01GX06-09, R06AX13, R06AX18, R06AX22, R06AX26-29, R06AE07, R06AE09, R01AD.
- C: based on Henriksen *et al.*'s algorithm [18] which by Stensballe *et al.* was shown a specificity in pediatric individuals on 0.80 and a sensitivity on 0.84[19]

Dates for clinical onset for all models for allergic rhinitis was defined as the first hospital contact with the specific diagnosis or the first prescription and for model A, the first date of prescription of any antihistamine will be coded as the date of clinical onset.

Asthma

Asthma is defined based on three different models—all only for children after age of 5 years:

- A: based on the Danish National Patient Register including ICD-10 codes:
    - o J45, J450, J451, J458, J459, J469 which had been validated in both children[21] and adults[45] with respectively specificities of 0.99 and 0.98.
- B: the most specific model from Moth *et al.* based on prescription data using DNPR with a specificity of 0.86[22]
    - o Prescription of:
        - ▪ Inhaled corticosteroid (ICS) (R03BA01, R03BA02, R03BA05)
            - • At least twice in 12 months
        - ▪ Inhaled short-acting beta2-agonist (SABA) (R03AC02, R03AC03, R03AC04)
            - • At least twice in 12 months

        - Inhaled long-lasting beta2-agonist (LABA) (R03AC12, RO3AC13, R03CC12)
            - At least twice in 12 months
        - Inhaled combination drugs of ICS and LABA (R03AK06, R03AK07)
            - At least twice in 12 months
        - Leukotriene antagonist (LTA) (R03DC03)
        - Rarely used anti-asthmatic drugs (R03BC01, R03AK03, R03AK04, R03BB01, R03BB04, R03DA04)
        - New asthmatic drugs since 2007 (ICS and ICS-LABA): R03B07, R03BA08, R03AK08, R03AK10, R03AK11, R03AK14
            - At least twice in 12 months
- C: a more specific model than B, using DNPR including at least 2 collections of the following specific anti-asthmatic drugs, a model not used before:
    - Inhaled corticosteroids (ICS) ATC-codes: R03AK06-14, R03BA
    - Leukotriene antagonists (LTA) ATC-code: R03DC

Children under 5 years of age was defined as persistent wheezing if criteria in model C for asthma is fulfilled, since asthma is not diagnosed in children in this age group. Clinical onset for all models for asthma or persistent wheezing was defined as the first hospital contact with the specific diagnosis in model A and the first prescription in model B and C.

Other definitions

Cystic fibrosis (CF) is defined based on hospital diagnosis as any ICD-code DE84x.

Cerebral Palsy (CP) is defined based on hospital diagnosis as any ICD-code DG80x

The atopic medication that are used for exclusion criteria for the secondary unexposed cohort to the atopic cohort is defined as dermatitis-drugs (D11AH), systemic steroids (H02), topical steroids (D07), inhalation steroids (R01AD, R03AK), nasal steroids (R03AL), or antihistamines (R06) with the first date of that medication included since criteria was no atopic medication prior to start of follow-up.

Delivery by caesarian section was based on mother's surgery diagnosis code KMCA from time of birth.

Prematurity was defined as gestational age less than 37 weeks and categorized from diagnosis code DP072 and DP073.

Season of birth was categorized based on birthdate in spring (March, April, and May), summer (June, July, and August), fall (September, October, and November) and winter (December, January, and February).

Family history of the outcome disease was for T1D based on parental T1D and for the atopic diseases based on full siblings since reliable parental atopic disease history was not available.

Definitions for the sensitivity methods:

i. an atopic dermatitis-cohort exposed or unexposed to T1D investigated later onset of asthma or allergic rhinitis
ii. mild and severe atopic dermatitis (defined as use of corticosteroids from group three or group four (D07AC, D07AD) and subsequent risk of T1D
iii. atopic dermatitis diagnosed by dermatologist or other specialties as exposure for T1D

iv. atopic dermatitis as both exposure and outcome for T1D with two new less strict or specific atopic dermatitis-algorithms defined as one prescription of D07 steroid lotion and based on Henriksen *et al*.'s algorithm[18] compared to the primary analysis
v. food allergy (defined as ICD-code DZ910x or DT780) alone or in a more severe type including prescription on adrenaline, C01CA24) as both exposure and outcome to T1D and as a sub analysis with combination of atopic dermatitis and food allergy
vi. asthma in a T2-high (defined as asthma + allergic rhinitis or food allergy) or T2-low (only asthma without any topical steroids used) as exposure for later T1D.

Supplementary tables

Supplementary table S1 – basic demographics of allergic rhinitis cohorts in model B and C

	**Allergic rhinitis (B) case (N=184,673)**	**Primary unexposed* (N=553,785)**	**Secondary unexposed (N=108,157)**
Sex (Female)	81,999 (44.4%)	245,990 (44.4%)	50,058 (46.3%)
Age at inclusion in study (Mean (SD))	8.97 (4.69)	8.97 (4.69)	6.39 (3.99)
Born by C-section	34,626 (18.7%)	89,645 (16.2%)	17,964 (16.6%)
Preterm birth	4,050 (2.2%)	11,665 (2.1%)	2,550 (2.4%)
Season of birth			
autumn	43,146 (23.4%)	129,496 (23.4%)	25,198 (23.3%)
spring	48,979 (26.5%)	146,892 (26.5%)	28,519 (26.4%)
summer	47,303 (25.6%)	141,814 (25.6%)	28,118 (26.0%)
winter	45,245 (24.5%)	135,583 (24.5%)	26,322 (24.3%)
At least one parent with T1D	3,682 (2.0%)	9,463 (1.7%)	1,964 (1.8%)
	**Allergic rhinitis (C) case (N=226,284)**	**Primary unexposed* (N=452,567)**	**Secondary unexposed (N=156,263)**
Sex (Female)	100,174 (44.3%)	200,348 (44.3%)	74,196 (47.5%)
Age at inclusion in study (Mean (SD))	6.92 (4.70)	6.92 (4.70)	4.37 (3.61)
Born by C-section	42,338 (18.7%)	73,644 (16.3%)	26,753 (17.1%)
Preterm Birth	5,275 (2.3%)	9,945 (2.2%)	3,941 (2.5%)
Season of birth			
autumn	53,232 (23.5%)	106,523 (23.5%)	37,121 (23.8%)
spring	59,638 (26.4%)	119,249 (26.3%)	40,403 (25.9%)
summer	58,108 (25.7%)	116,212 (25.7%)	41,158 (26.3%)
winter	55,306 (24.4%)	110,583 (24.4%)	37,581 (24.0%)
At least one parent with T1D	4,457 (2.0%)	7,533 (1.7%)	2,745 (1.8%)

Abbreviations: SD, standard deviation, C-section, Caesarian section, T1D, Type 1 Diabetes

*The number of random age- and sex-matched individuals per case were for allergic rhinitis in model B three and for allergic rhinitis in model C two.

Supplementary Table S2 – basic demographics of asthma cohorts in model B and C and persistent wheezing in model C

	**Asthma (B) Case (N=89,807)**	**Primary unexposed* (N=538,842)**	**Secondary unexposed (N=117,371)**
Sex (Female)	37,711 (42.0%)	226,266 (42.0%)	53,767 (45.8%)
Age at inclusion in study (Mean (SD))	8.49 (3.57)	8.49 (3.57)	7.31 (2.70)
Born by C-section	18,284 (20.4%)	88,640 (16.5%)	18,704 (15.9%)
Preterm Birth	2,996 (3.3%)	11,054 (2.1%)	1,881 (1.6%)
Season of birth			
autumn	21,757 (24.2%)	130,656 (24.2%)	27,941 (23.8%)
spring	22,748 (25.3%)	136,618 (25.4%)	30,034 (25.6%)
summer	23,538 (26.2%)	141,103 (26.2%)	30,864 (26.3%)
winter	21,764 (24.2%)	130,465 (24.2%)	28,532 (24.3%)
At least one parent with T1D	1,828 (2.0%)	9,490 (1.8%)	2,190 (1.9%)
	**Asthma (C) Case (N=76,682)**	**Primary unexposed* (N=460,092)**	**Secondary unexposed (N=112,281)**
Sex (Female)	31,414 (41.0%)	188,484 (41.0%)	50,154 (44.7%)
Age at inclusion in study (Mean (SD))	8.44 (3.47)	8.44 (3.47)	7.32 (2.62)
Born by C-section	15,570 (20.3%)	75,599 (16.4%)	17,416 (15.5%)
Preterm Birth	2,468 (3.2%)	9,065 (2.0%)	1,634 (1.5%)
Season of birth			
autumn	18,614 (24.3%)	111,774 (24.3%)	26,762 (23.8%)
spring	19,439 (25.4%)	116,764 (25.4%)	28,997 (25.8%)
summer	20,057 (26.2%)	120,296 (26.1%)	29,428 (26.2%)
winter	18,572 (24.2%)	111,258 (24.2%)	27,094 (24.1%)
At least one parent with T1D	1,579 (2.1%)	8,212 (1.8%)	2,175 (1.9%)
	**Persistent wheezing (C) Case (N=144,789)**	**Primary unexposed* (N=723,921)**	**Secondary unexposed (N=259,754**
Sex (Female)	56,732 (39.2%)	283,658 (39.2%)	100,448 (38.7%)
Age at inclusion in study (Mean (SD))	1.91 (1.08)	1.91 (1.08)	1.70 (0.978)
Born by C-section	33,251 (23.0%)	128,609 (17.8%)	46,879 (18.0%)
Preterm Birth	8,379 (5.8%)	22,153 (3.1%)	6,842 (2.6%)
Season of birth			
autumn	36,472 (25.2%)	182,324 (25.2%)	64,347 (24.8%)
spring	35,663 (24.6%)	178,342 (24.6%)	65,162 (25.1%)
summer	38,230 (26.4%)	191,141 (26.4%)	68,750 (26.5%)
winter	34,424 (23.8%)	172,114 (23.8%)	61,495 (23.7%)
At least one parent with T1D	2,836 (2.0%)	11,184 (1.5%)	4,491 (1.7%)

Abbreviations: SD, standard deviation, C-section, Caesarian section, T1D, Type 1 Diabetes

*The number of random age- and sex-matched individuals per case were for asthma in model B and C six, and for persistent wheezing only two.

Supplementary Table S3 – Effects of Type 1 Diabetes on later atopic disease compared to secondary unexposed cohorts

	IR_A [95%CI]	IR_B [95%CI]	HR [95% CI]	P_{raw}	P_{corr}	N	Events
Risk of atopic dermatitis							
Unadjusted model	0.71 [0.45;1.13]	0.99 [0.67;1.45]	0.79 [0.41;1.50]	0.462	1.000	8,375	38
Adjusted model§			0.91 [0.45;1.85]	0.801	1.000	8,175	36
Risk of allergic rhinitis							
Unadjusted model A	0.95 [0.64;1.41]	1.60 [1.18;2.17]	0.57 [0.34;0.97]	0.039	0.235	8,375	60
Adjusted model§ A			0.59 [0.34;1.03]	0.065	0.260	8,175	59
Unadjusted model B	30.39 [28.18;32.76]	29.12 [27.02;31.40]	0.94 [0.81;1.08]	0.364	0.727	7,785	736
Adjusted model§ B			0.89 [0.76;1.03]	0.121	0.362	7,588	724
Unadjusted model C	38.74 [36.17;41.49]	41.68 [39.05;44.49]	0.84 [0.72;0.98]	0.029	0.235	7,346	647
Adjusted model§ C			0.90 [0.76;1.06]	0.206	0.494	7,156	632
Risk of asthma							
Unadjusted model A	3.23 [2.60;4.03]	3.72 [3.05;4.54]	0.78 [0.57;1.06]	0.107	0.341	8,370	166
Adjusted model§ A			0.88 [0.63;1.23]	0.445	1.000	8,170	161
Unadjusted model B	13.05 [11.68;14.59]	15.93 [14.43;17.58]	0.63 [0.50;0.79]	< 0.001	0.001	8,009	322
Adjusted model§ B			0.78 [0.61;1.00]	0.049	0.195	7,815	313
Unadjusted model C	11.06 [9.81;12.48]	11.81 [10.54;13.23]	0.74 [0.58;0.94]	0.016	0.084	8,087	262
Adjusted model§ C			0.93 [0.72;1.22]	0.620	1.000	7,889	256
Unadjusted model persistent wheezing	2.46 [1.75;3.46]	6.13 [4.98;7.54]	0.41 [0.27;0.61]	< 0.001	< 0.001	7,356	122
Adjusted§ persistent wheezing			1.21 [0.79;1.86]	0.378	1.000	7,173	120

Raw incidence rates (IR) shown per 1000 person-year for respectively T1D-exposed cohort (A) and the secondary unexposed cohort as control cohort (B), Hazard ratio (HR) from Cox regressions is based on exposed case cohort compared to the secondary unexposed cohort (individuals with cerebral palsy). The raw p-values are shown as P_{raw}, where P_{corr} are familywise corrected by Benjamini-Hochberg

§Adjusted models are adjusted for age group, sex, born by Caesarian section, prematurity, season of birth and relevant family history (sibling with asthma for models of risk of asthma etcetera).

Supplementary Table S4 – Effects of atopic disease on later type 1 diabetes compared to secondary unexposed cohorts

Table S4	IR_A [95%CI]	IR_B [95%CI]	HR [95% CI]	P_{raw}	P_{corr}	N	Events
Atopic dermatitis and risk of type 1 diabetes							
Unadjusted model	0.27 [0.20;0.35]	0.38 [0.34;0.43]	0.78 [0.57;1.05]	0.098	0.196	101,680	365
Adjusted model§			0.72 [0.53;0.98]	0.035	0.141	101,314	365
Allergic rhinitis and risk of type 1 diabetes							
Unadjusted model A	0.48 [0.38;0.60]	0.42 [0.37;0.48]	1.38 [1.04;1.82]	0.024	0.271	61,249	239
Adjusted model§ A			1.33 [1.01;1.76]	0.045	0.271	60,963	239
Unadjusted model B	0.36 [0.33;0.40]	0.54 [0.49;0.59]	0.92 [0.80;1.06]	0.256	0.615	288,513	745
Adjusted model§ B			0.90 [0.78;1.05]	0.170	0.588	287,008	745
Unadjusted model C	0.34 [0.32;0.37]	0.45 [0.42;0.49]	0.95 [0.85;1.07]	0.426	0.852	379,834	1,154
Adjusted model§ C			0.92 [0.82;1.04]	0.196	0.588	378,148	1,153
Asthma and risk of type 1 diabetes							
Unadjusted model A	0.35 [0.28;0.43]	0.60 [0.53;0.66]	0.85 [0.65;1.12]	0.248	0.567	90,466	263
Adjusted model§ A			0.85 [0.65;1.11]	0.227	0.567	89,713	262
Unadjusted model B	0.43 [0.38;0.49]	0.53 [0.49;0.58]	1.11 [0.95;1.30]	0.193	0.567	204,896	659
Adjusted model§ B			1.08 [0.92;1.26]	0.361	0.722	203,392	658
Unadjusted model C	0.42 [0.37;0.48]	0.48 [0.44;0.52]	1.15 [0.98;1.36]	0.092	0.492	187,134	594
Adjusted model§ C			1.13 [0.95;1.33]	0.168	0.567	185,725	592
Unadjusted model persistent wheezing	0.38 [0.35;0.41]	0.35 [0.33;0.37]	1.18 [1.06;1.31]	0.002	0.029	402,185	1,558
Adjusted model§ persistent wheezing			1.15 [1.03;1.28]	0.009	0.074	401,213	1557

Raw incidence rates (IR) shown per 1000 person-year for respectively T1D-exposed cohort (A) and the secondary unexposed cohort as control cohort (B), Hazard ratio (HR) from Cox regressions is based on exposed case cohort compared to the secondary unexposed cohort (individuals without use of atopic medicine). The raw p-values are shown as P_{raw}, where P_{corr} are familywise corrected by Benjamini-Hochberg

§Adjusted models are adjusted for age group, sex, born by Caesarian section, prematurity, season of birth and relevant family history (parents with type 1 diabetes).

Supplementary Table S5 – Results from the sensitivity analyses

	HR	95% CI	P-value
**(i) atopic dermatitis-cohort exposed or unexposed to T1D**			
Risk of later rhinitis	*Assumptions for Cox regression not fulfilled, but both log-rank test insignificant*		
Risk of later asthma			
**(ii) mild and severe atopic dermatitis and risk of T1D in adjusted analysis**			
Effect of case (atopic dermatitis)	1.02	[0.70;1.47]	0.929
Effect of severe atopic dermatitis	1.10	[0.88;1.36]	0.404
Effect of interaction of case and severe atopic dermatitis	0.55	[0.30;1.01]	0.053
**(iii) atopic dermatitis diagnosed by dermatologist or other specialty**			
Risk of later T1D	*Cox regression results in infinite values and log-rank test insignificant*		
**(iv)-a atopic dermatitis as one prescription of steroid lotion**			
Risk of T1D	1.17	[1.09:1.26]	<0.001
Risk of atopic dermatitis	*Assumptions for Cox regression not fulfilled, but log-rank test with p< 0.0001 and positive association*		
**(iv)-b atopic dermatitis as Henriksen et al algorithm**			
Risk of T1D	0.92	[0.84;1.01]	0.073
Risk of atopic dermatitis after T1D	1.22	[1.05;1.40]	0.008
**(v)-a food allergy as exposure and outcome**			
Risk of T1D	0.79	[0.52;1.21]	0.280
Risk of food allergy after T1D	0.78	[0.47;1.30]	0.335
**(v)-a food allergy and atopic dermatitis**			
Risk of T1D	0.69	[0.28;1.72]	0.429
**(vi) Asthma stratified in T2-high or T2-low models for risk of T1D compared to healthy controls**			
Effect of T2-low<br>Effect of T2-middle *for asthma as algorithm A*<br>Effect of T2-high	0.64<br>0.85<br>1.14	[0.35;1.16]<br>[0.61;1.18]<br>[0.77;1.67]	0.142<br>0.326<br>0.514
Effect of T2-low<br>Effect of T2-middle *for asthma as algorithm B*<br>Effect of T2-high	1.18<br>1.15<br>1.23	[0.92;1.51]<br>[0.98;1.36]<br>[0.89;1.70]	0.184<br>0.092<br>0.210
Effect of T2-low<br>Effect of T2-middle *for asthma as algorithm C*<br>Effect of T2-high	1.16<br>1.15<br>1.18	[0.88;1.52]<br>[0.96;1.38]<br>[0.83;1.67]	0.295<br>0.122<br>0.350

Abbreviations: HR, hazard ratio, CI, confidence interval, T1D, type 1 diabetes

Supplementary figures

Supplementary Figure S1 – Directed Acyclic Graphs

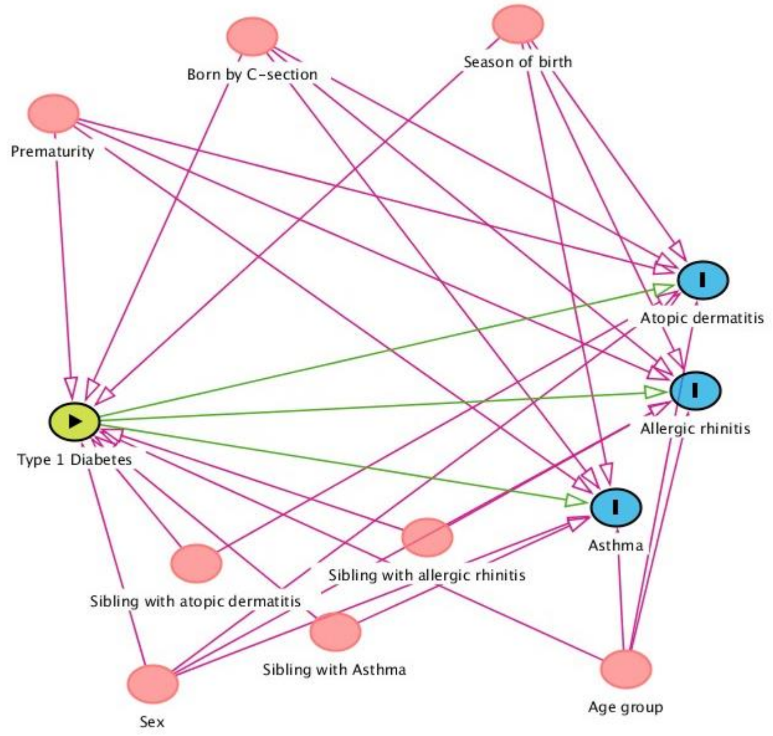

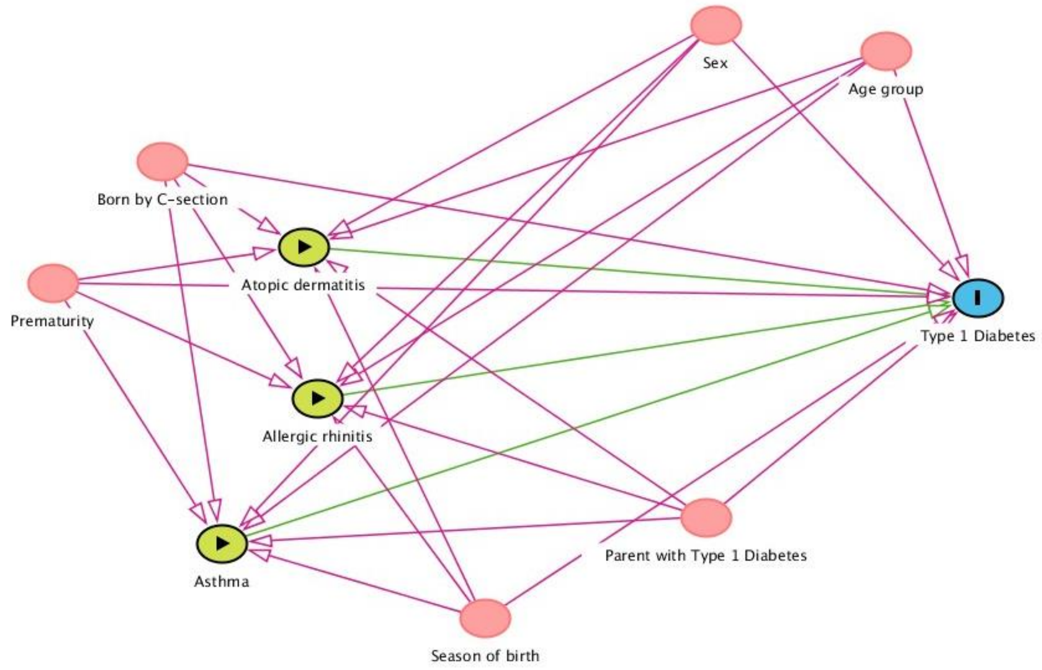

Directed acyclic graphs (DAG) which do show the green exposure, the blue outcomes, and the red potential confounders which are adjusted for in multivariable analyses. Made at dagitty.net

Supplementary Figure S2 - T1D as exposure

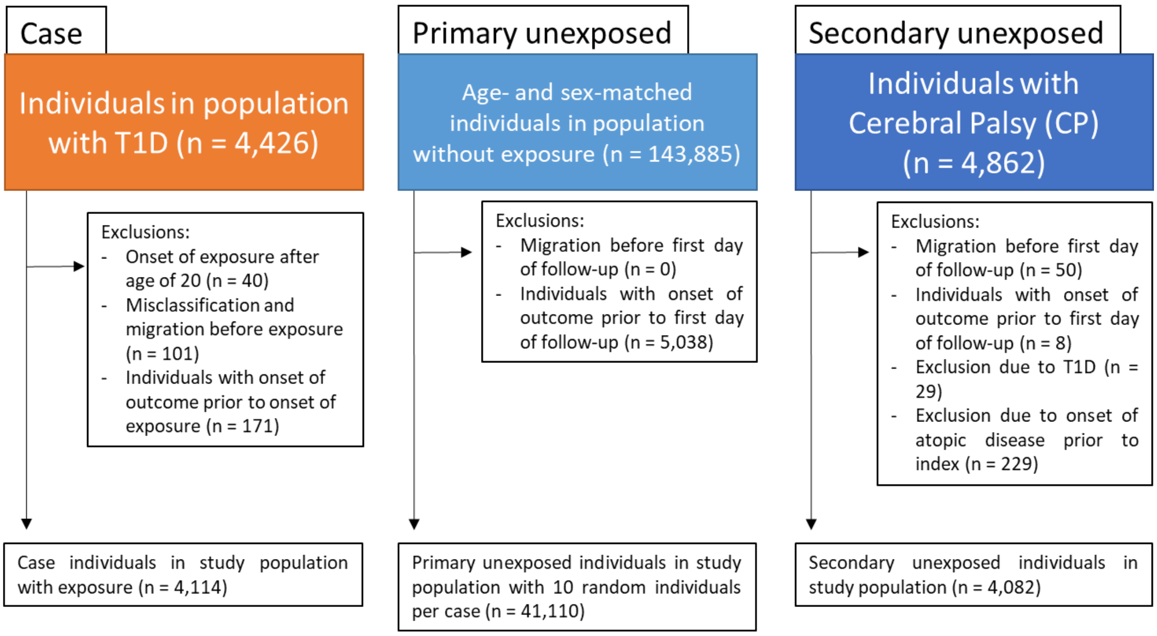

Flowchart for how case cohort, primary and secondary unexposed cohorts are derived with Type 1 Diabetes (T1D) as exposure.

Supplementary Figure S3 – Atopic dermatitis as exposure

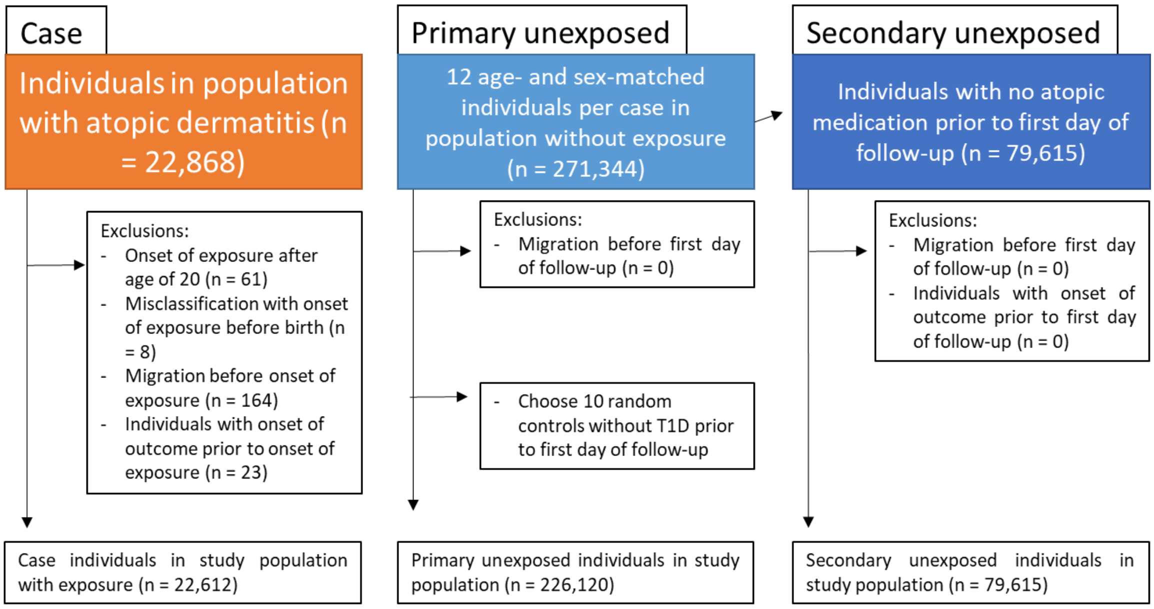

Flowchart for how case cohort, primary and secondary unexposed cohorts are derived with atopic dermatitis as exposure.

Supplementary Figure S4 – Allergic rhinitis A as exposure

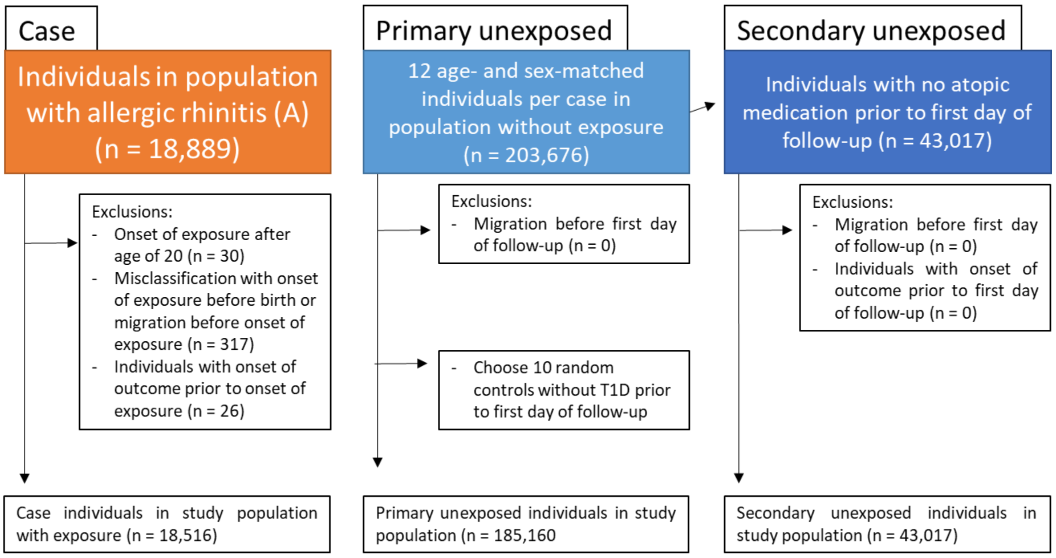

Flowchart for how case cohort, primary and secondary unexposed cohorts are derived with allergic rhinitis defined by algorithm A as exposure.

Supplementary Figure S5 – Allergic rhinitis (B) as exposure

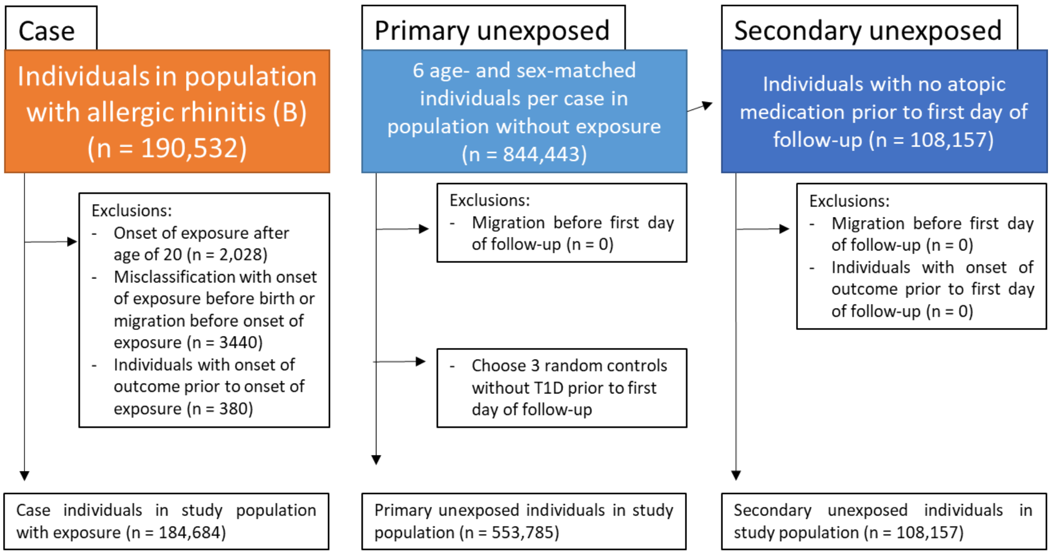

Flowchart for how case cohort, primary and secondary unexposed cohorts are derived with allergic rhinitis defined by algorithm B as exposure.

Supplemental figure S6– Allergic rhinitis (C) as exposure

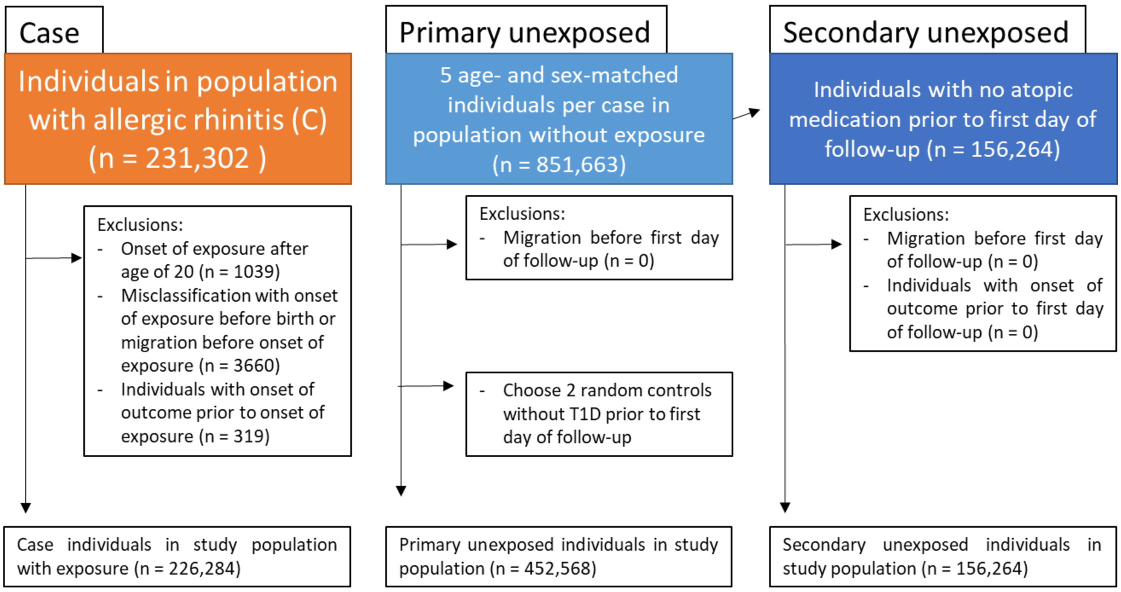

Flowchart for how case cohort, primary and secondary unexposed cohorts are derived with allergic rhinitis defined by algorithm C as exposure.

Supplemental figure S7 – Asthma (A) as exposure

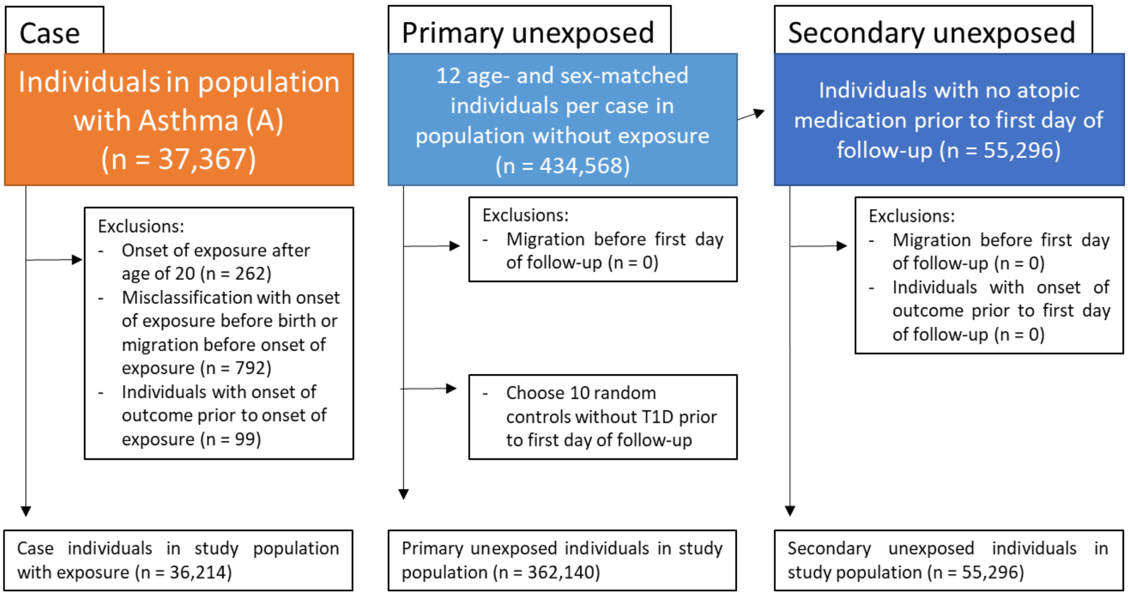

Flowchart for how case cohort, primary and secondary unexposed cohorts are derived with asthma defined by algorithm A (only with onset after age of five years) as exposure.

Supplemental figure S8 – Asthma (B) as exposure

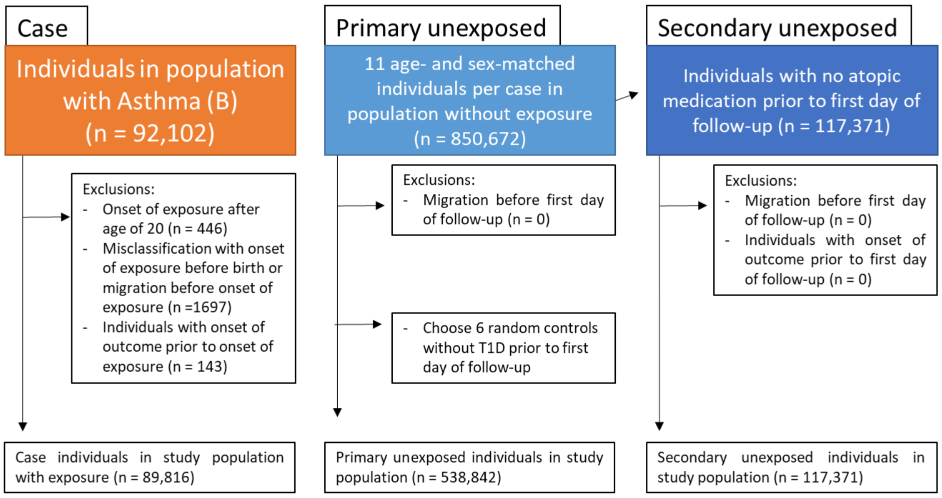

Flowchart for how case cohort, primary and secondary unexposed cohorts are derived with asthma defined by algorithm B (only with onset after age of five years) as exposure.

Supplemental figure S9 – Asthma (C) as exposure

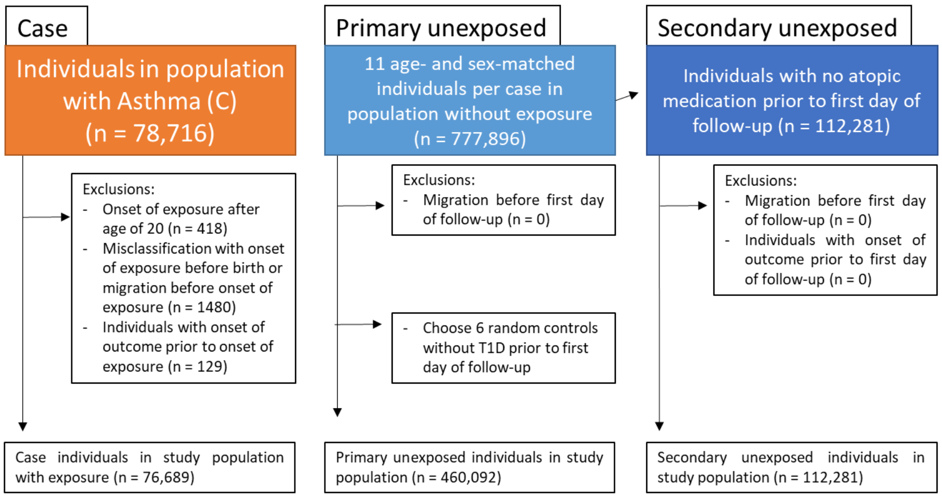

Flowchart for how case cohort, primary and secondary unexposed cohorts are derived with asthma defined by algorithm C (only with onset after age of five years) as exposure.

Supplemental figure S10 – Persistent wheezing as exposure

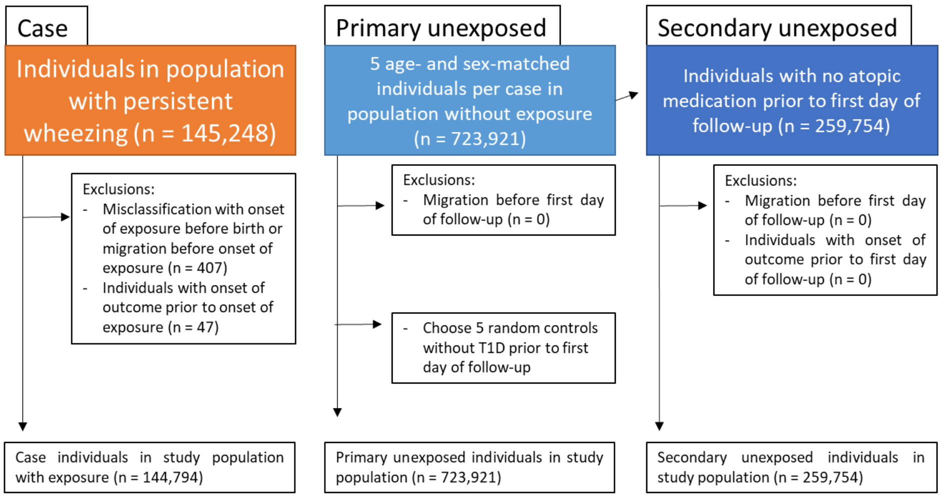

Flowchart for how case cohort, primary and secondary unexposed cohorts are derived with persistent wheezing defined by algorithm C with age at onset under or equal to five years as exposure.
